# Why Child Health and Mortality Prevention Surveillance?

**DOI:** 10.1093/cid/ciz542

**Published:** 2019-10-09

**Authors:** Scott F Dowell, Anita Zaidi, Penny Heaton

**Affiliations:** 1 Bill & Melinda Gates Foundation, Seattle, Washington; 2 Gates Medical Research Institute, Boston, Massachusetts

**Keywords:** child mortality, surveillance, pathology, disease burden, global health

## Abstract

Recognizing the need for better primary data on the causes of global child mortality, the Bill & Melinda Gates Foundation made an unusually long funding commitment toward a surveillance system using pathology to identify opportunities to prevent child deaths and promote equity.

The Bill & Melinda Gates Foundation is dedicated to the idea that every child deserves the chance for a healthy and productive life. By 2015, progress in reducing childhood mortality had been remarkable, declining to <6 million deaths per year from 12 million in 1990. But mortality remained unacceptably high in much of Africa and South Asia, where the risk that a child would die before their fifth birthday remained some 10-fold higher than in wealthier countries [1].

Perhaps most frustrating for a data-driven organization such as ours was that we had very poor information about what was killing these children. Only a tiny fraction, <5% of child deaths in the highest-mortality countries, were medically certified [2]. What little information we had about specific causes came largely from verbal autopsy studies—interviews with a parent some months later about the circumstances surrounding the death that were used to assign a probable underlying cause from a series of broad syndromic categories.

Beginning in 2015, we committed to more definitive evidence, generated through surveillance for the causes of child mortality. We sought data from a representative sample of the highest-mortality places in Africa and South Asia, and methods to be as close to the gold standard of autopsy as possible. Minimally invasive tissue sampling (MITS) was a technique to provide needle biopsy pathology samples that was adapted for use in developing countries by the ISGlobal research group in Barcelona and Mozambique. But there was deep uncertainty about whether it was possible to do MITS at scale in high-risk populations, and skeptics were many. We were told that parents would not accept autopsies and that there were not nearly enough pathologists.

To succeed, we knew we would need to work with the world’s most capable partners. The International Association of National Public Health Institutes provided leadership and legitimacy, the Centers for Disease Control and Prevention world-class surveillance and laboratory expertise, and the London School of Hygiene and Tropical Medicine long experience innovating in high-risk countries to address disease burden; all were coordinated by Emory University’s Global Health Institute in partnership with some of the leading pediatricians and investigators in Africa and South Asia. A representative sample of 7 initial sites was selected from >50 applicants. We began with an analysis of the child mortality cause distribution based on available Global Burden of Disease data from all countries in Africa and South Asia, constructed a dendrogram from the known cause distribution, and selected at least 1 site from each of the major branches (high malaria areas, low human immunodeficiency virus/high perinatal, etc). We required that all sites meet minimum criteria, including an under-5 mortality rate exceeding 50 per 1000 live births and a willingness to engage deeply with communities to attempt MITS and share anonymized data as a global public good.

Pilot studies in South Africa and Bangladesh were instructive. There was a much higher rate of acceptance of MITS than anticipated in South Africa, with both mothers and fathers asking for the results of testing on their deceased infants.(see Madhi et al in this supplement). A similar pilot in Bangladesh was discontinued because of slow progress on necessary clearances and low enrollment. Elements of success included strong local leadership and established relationships with hospital staff and community.

Now the availability of MITS is beginning to change the way we assess and analyze the causes of death in children in high-mortality settings. High rates of consent by parents across the first 5 sites has resulted in more complete case information packages than anticipated. The evaluation of each death includes a review of the clinical story from the verbal autopsy, supplemented by the clinical record for those who were hospitalized, with extensive postmortem laboratory testing for all. Several hundred test results on each death are supplemented most importantly by the pathologic examination of multiple tissues using routine stains and a broad array of specific immunohistochemical tests that places any detected organisms in the context of the tissue and immune response. An average of 90 pages of summarized results on each death is provided to a panel of physicians to complete the death certificate. Standard World Health Organization rules for coding and certification, with benefit from this unprecedented level of detail, allow for the most definitive possible assignment of the full causal chain for each death. The hundreds, and soon thousands, of MITS-informed deaths can be used to calibrate other methods for assigning cause, such as verbal autopsy and medical certification, to broadly improve cause of death assignment for children across the highest-mortality regions. Incorporation of these results into the Global Burden of Disease and similar global estimation efforts should improve the accuracy of global mortality statistics.

Significant challenges remain. We still lack substantial numbers of MITS from South Asia, a region with lower rates of mortality than Africa but much larger populations and therefore an important contributor to the global burden of childhood deaths. Deaths that occur at home or in the community are an important challenge, and only 15%–20% of currently captured deaths occurred at home. Capturing a representative sample of these will be necessary to provide an accurate picture of the causal distribution. In addition, the extensive testing provides both a strength of the Child Health and Mortality Prevention Surveillance (CHAMPS) method and a challenge, in that ordering and prioritizing the events in the causal chain requires some novel methodological approaches.

Expanding the number of CHAMPS sites modestly, obtaining additional samples from South Asia, and increasing the number of investigators using MITS to identify causes of death continue to be objectives to increase the representativeness of the data. Recognizing that the value of good surveillance systems increases over time, the foundation is committed to sustain CHAMPS over the long term. We are interested in building on the CHAMPS platform by testing new approaches such as machine learning to identify normal and abnormal pathology and improve and standardize the cause of death attribution, and pathogen genomic sequencing to identify strain differences and characterize antimicrobial resistance patterns.

Teams across the Gates Foundation are beginning to use CHAMPS early data to shape and refine our impressions of some of the important unaddressed causes for childhood mortality (review current data at champshealth.org). In addition to confirming the important roles of perinatal deaths, pneumonia, diarrhea, and malaria, CHAMPS data permit a deeper dive of these syndromic pieces of the etiological pie. We can begin to quantify the roles of nosocomial gram-negative pathogens in vulnerable preterm infants, group B streptococci in perinatal deaths, systemic obstetrical challenges in birth asphyxia, the co-occurrence of cerebral malaria with certain agents of bacterial sepsis, and the magnitude of respiratory syncytial virus (RSV) deaths among infants <6 months of age.

With this additional level of specificity on the causes of death, more directed interventions become feasible, beginning at the local level. One site has already addressed an MITS-confirmed problem with nosocomial sepsis by completing a systematic external review of infection control practices and instituted an intensified push to reduce deaths by modifying the preparation of intravenous medications, enforcing barrier precautions, and hiring dedicated infection control staff. Early infant RSV deaths across the sites have prompted a reassessment of the potential value of a maternal RSV vaccine. We anticipate that as the data begin to grow in numbers, representativeness, and value, decision makers from outside the foundation also will use them to direct more effective interventions to accelerate reductions in childhood mortality.

As pediatricians, we value the opportunity to know the causes of childhood mortality more definitively than ever and to use that information to accelerate reductions in childhood mortality. In the end, CHAMPS should help to advance global health equity—by bringing the world’s most sophisticated postmortem testing to childhood deaths in the world’s most vulnerable regions, it will advance our understanding of preventable causes of death and our ability to address them—so that all children of tomorrow have an equal chance for a healthy and productive life.

## Notes

***Financial support.*** This work was supported by the Bill & Melinda Gates Foundation [OPP1126780].

***Supplement sponsorship.*** This supplement is sponsored by the Emory Global Health Institute and the Bill & Melinda Gates Foundation.

***Potential conflicts of interest.*** S.D. and A.Z. are employed by the funder of the CHAMPS (Child Health and Mortality Prevention Surveillance) program. All other authors report no potential conflicts of interest. All authors have submitted the ICMJE Form for Disclosure of Potential Conflicts of Interest. Conflicts that the editors consider relevant to the content of the manuscript have been disclosed.
